# Prognostic indicators of new onset atrial fibrillation in patients with acute coronary syndrome

**DOI:** 10.1002/clc.23363

**Published:** 2020-04-14

**Authors:** Hengliang Zhang, Pingshuan Dong, Xvming Yang, Laijing Du, Ke Wang, Peng Yan, Huifeng Zhang, Tengfei Wang, Xikun Zhao, Tengfei Guo

**Affiliations:** ^1^ The First Affiliated Hospital, and College of Clinical Medicine of Henan University of Science and Technology Luoyang China

**Keywords:** ACS, AF, CK‐MB, LVEF, NT‐proBNP

## Abstract

**Background:**

This study aims to estimate prognostic indicators of new onset atrial fibrillation (AF) in patients with acute coronary syndrome (ACS) through 3 to 5 years of follow‐up.

**Hypothesis:**

For patients with ACS, some prognostic indicators can be used to predict new onset AF.

**Methods:**

The Improving Care for Cardiovascular Disease in China‐ACS (CCC‐ACS) program was launched in 2014 by a collaborative initiative of the American Heart Association and Chinese Society of Cardiology. We enrolled 866 patients with ACS in a telephone follow‐up program. We inquired about each patient's general health and invited each patient to our hospital for further consultation. We also performed ambulatory electrocardiography and other relevant examinations.

**Results:**

A total of 743 ACS patients were included in the study. After 3 to 5 years, 50 (0.67%) patients developed AF. In multivariable Cox models adjusting for AF risk factors in ACS patients, we found that NT‐proBNP [hazard ratio (HR) 2.625, 1.654‐4.166, *P* < .05], creatine kinase‐MB (CK‐MB) (HR 4.279, 1.887‐9.703, *P* < .05), and left ventricular ejection fraction (LVEF) (HR 0.01, 0.001‐0.352, *P* < .05) were significantly associated with AF receiver operating characteristic (ROC) curves were used to determine a cutoff level for AF screening. NT‐proBNP using a cutoff of 1705 ng/L resulted in a sensitivity of 58% and a specificity of 89.8%. CK‐MB using a cutoff of 142.5 ng/L resulted in a sensitivity of 73.3% and a specificity of 58.3%.

**Conclusion:**

For patients with ACS, NT‐proBNP, CK‐MB, and LVEF have a considerable prognostic value for predicting whether AF would be detected during follow‐up.

## 1 INTRODUCTION

Atrial fibrillation (AF) is the most common arrhythmia in adults, with an incidence of 3%, which gradually increases with age. The incidence among individuals over 80 is over 13%.^1^

Patients with acute coronary syndrome (ACS) are more likely to have arrhythmias such as AF and other arrhythmias after myocardial injury than those without ACS. AF patients are more likely to develop ACS due to coronary artery embolism than those without AF.^2^ AF and ACS affect each other, and this can lead to the rapid development of the disease and eventually poor prognosis. Therefore, early detection and intervention in AF and ACS patients are particularly important.

Some biomarkers have been confirmed to be associated with the occurrence and development of AF,^3^, ^4^ and it may be possible to reduce the complications of AF through more intensive screening.

We used a multimarker approach with eight indexes, including heart failure markers, myocardial infarction markers, plasma glucose, creatinine, low‐density lipoprotein cholesterol (LDL‐C), and left ventricular ejection fraction (LVEF) to determine whether these indicators can predict the occurrence and development of AF.

## 2 METHODS

### 2.1 Study populations

The Improving Care for Cardiovascular Disease in China‐ACS (CCC‐ACS) project is a national registry and quality improvement study with a database that focuses on the quality of treatment and care for ACS.

The project was launched in 2014 by collaborative initiative of the American Heart Association and Chinese Society of Cardiology. Because the design and methods of the CCC‐ACS project have been published in a previous report, we will not go into detail here.^5^

Our study was based on the CCC‐ACS project and included all of the ACS patients in the First Affiliated Hospital of Henan University of Science and Technology from January 2014 to December 2017. During this period, we collected baseline data, laboratory tests, electrocardiograms, and echocardiography covering all of the ACS patients. All of the patients with acute ST segment elevation myocardial infarction, or non‐ST segment elevation myocardial infarction, or unstable angina pectoris were included in ACS. Patients who had experienced AF before ACS were excluded. If there was no contraindication, all of the patients were given dual antiplatelet, aspirin and clopidogrel, or tegrarol and statins. If the drug had not been taken orally before, it must be given a load. According to the clinical situation of patients, angiotensin converting enzyme inhibitors and beta‐blocker drugs were given on time. From September 2019 to October 2019, these patients were followed up intensively and asked to go to our outpatient clinic for echocardiography, ambulatory electrocardiography, and other related examinations. The primary end point was defined as the first detection of AF during follow‐up.

All of the participants received written informed consent, which was approved by the ethics committee of the First Affiliated Hospital of Henan University of Science and Technology.

### 2.2 Baseline measurements and biochemical analysis

At first hospitalization, participants' gender, age, smoking habits, systolic blood pressure, heart rate, history of myocardial infarction, and history of percutaneous coronary intervention (PCI) were recorded. AF was diagnosed on an ECG obtained using a Holter monitor or hospital records. Whether the ECG recording indicated AF was determined by a cardiologist. Smoking history was defined as smoking at least once every day for at least 1 year. Diabetes is defined as glycosylated hemoglobin greater than 6% or long‐term use of oral drugs or subcutaneous insulin injection to control blood sugar. Quality control of laboratory indicators and definition of medical history standard were performed with reference to the CCC Project.

### 2.3 Statistical analysis

Baseline variables are presented as mean, SD, and percentages. We analyze normality of all of the measurement data. If a variable does not conform to normal distribution, log transformation is carried out and represented by the median and interquartile range. Cox proportional hazards regression models were tested for every marker. Results were adjusted for cardiovascular risk factors comprising of gender, age, smoking habits, systolic blood pressure, heart rate, and history of type 2 diabetes, history of heart failure, history of myocardial infarction, and history of PCI. All of the statistical testes and confidence intervals were two‐sided, and *P* < .05 was used to identify statistically significant results. Statistical analysis of data was performed using SPSS19 software.

## 3 RESULT

We enrolled 866 patients with ACS and conducted telephone follow‐up. We were able to collect clinical data of 743 patients, including 316 (42.5%) patients with acute ST segment elevation myocardial infarction, 136 (18.3%) patients with non‐ST segment elevation myocardial infarction, and 291 (39.2%) patients with unstable angina pectoris. The baseline characteristics of these patients are shown in Table 1. The average age of the participants was 62 ± 12 years, 78.5% of whom were men. One hundred and twenty‐six (14.5%) patients had clinical symptoms and signs of heart failure at the time of admission. Twenty‐five (3.4%) patients had moderate to severe valve regurgitation. The changes of biomarkers of myocardial infarction and heart failure over time are listed in Table 2. NT‐proBNP and other clinical data, which do not conform to the normal distribution, were naturally logarithmically transformed (log_e_). Up to the end of the follow‐up, 50 patients were found to have developed new AF (0.67%).

**TABLE 1:** Baseline characteristics

Clinical	N = 743
Age (y)	62 ± 12
Sex (man)	583(78.5%)
Heart rate (bpm)	76 ± 16
Systolic blood pressure (mmHg)	127 ± 26
Current smoking	361(48.6%)
Diabetes	132(17.8%)
History of heart failure	76(10.2%)
History of myocardial infarction	48(6.5%)
History of PCI	44(5.9%)
Hypertension	346(46.6%)
Troponin (ng/mL)	2.60 (0.33, 11.00)
NT‐proBNP (pg/mL)	321.0 (123.0, 773.5)
CK‐MB (IU/L)	121 (159, 226)
Creatinine (umol/L)	89.0 (80.5, 102.0)
Hb (g/L)	124 (109, 133)
Fasting blood glucose (mmol/L)	5.5 (4.7, 7.0)
LDL‐C (mmol/L)	2.70 (2.11, 3.29)
LVEF (%)	51 (45, 58)

*Notes:* Continuous values are represented by mean ± SD. Non‐normal distribution values are represented by median (25th, 75th).

Abbreviations: Hb, hemoglobin; LDL‐C, low‐density lipoprotein cholesterol; LVEF, left ventricular ejection fraction; NT‐proBNP, N‐terminal pro‐brain natriuretic peptide; PCI, percutaneous coronary intervention.

**TABLE 2:** Fluctuation of biomarkers over time

	Admission	24 hours	3 months
Troponin (ng/mL)	2.60 (0.33, 11.00)	2.80 (0.22, 11.80)	/
NT‐proBNP (pg/mL)	321.0 (123.0, 773.5)	635.0 (256.0, 1056.0)	169.0 (80.0, 472.0)
Ck‐MB (IU/L)	121 (59, 226)	136 (32, 218)	13 (9, 18)
Hb (g/L)	124 (109, 133)	121 (103, 136)	123 (112, 138)
LVEF (%)	51 (45, 58)	42 (38, 53)	50 (41, 56)
LA diameter (mm)	30 (27, 35)	31 (26, 37)	33 (23, 41)

Abbreviations: Hb, hemoglobin; LA, left atrial; LVEF, left ventricular ejection fraction; NT‐proBNP, N‐terminal pro‐brain natriuretic peptide.

To identify the risk factors of new onset AF, we analyzed each possible factor using a Cox proportional hazards regression model. In the multivariate model analysis, we set age, gender, medical history, and other information as the baseline data, and then NT proBNP, TnI, CK‐MB, creatinine, hemoglobin, LVEF, blood glucose, and blood lipid were introduced into the model. The HR of each indicator is shown in Table 3. We found that the value of NT‐proBNP (HR 2.625, 1.654‐4.166, *P* < .05), CKMB (HR 4.279, 1.887‐9.703, *P* < .05), and LVEF (HR 0.01, 0.001‐0.352, *P* < .05) were significantly closely associated with AF at the beginning of admission (Table 3). After 3 months, only the LVEF (HR 0.03, 0.005‐0.452, *P* < .05) was significantly closely associated with AF.

**TABLE 3:** Multivariable‐adjusted proportional hazards regression models for AF

Adjustment factors	HR	*P* values
Baseline+log_e_ NT‐proBNP	2.625 (1.654‐4.166)	<.05
Baseline+log_e_ TnI	1.236 (0.820‐1.863)	.31
Baseline+log_e_ CK‐MB	4.279 (1.887–9.703)	<.05
Baseline+log_e_ Cr	0.263 (0.026‐2.689)	.263
Baseline+log_e_ Hb	1.856 (0.013‐272.729)	.808
Baseline+log_e_ glucose	0.852 (0.121‐6.014)	.872
Baseline+log_e_ LDL‐C	0.440 (0.059‐3.259)	.421
Baseline+log_e_ LVEF	0.010 (0.000‐0.352)	<.05

*Notes:* Baseline included in gender, age, smoking habits, systolic blood pressure, heart rate, history of diabetes, history of heart failure, history of myocardial infarction, and history of PCI.

Abbreviations: AF, atrial fibrillation; CK‐MB, creatine kinase‐MB; Cr, creatinine; Hb, hemoglobin; LDL‐C, low‐density lipoprotein cholesterol; LVEF, left ventricular ejection fraction; NT‐proBNP, N‐terminal probrain natriuretic peptide; TnI, troponin I.

**FIGURE 1:**
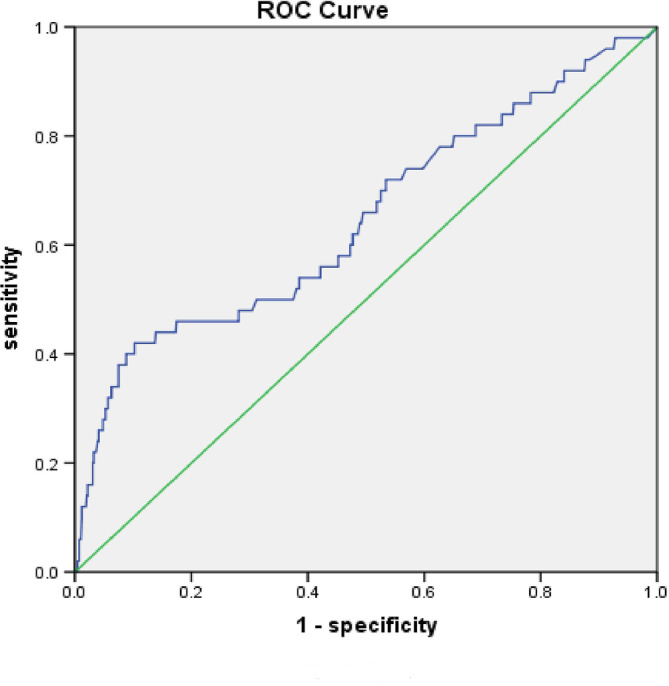
ROC curve depicting detection of new atrial fibrillation as a function of NT‐proBNP

**FIGURE 2:**
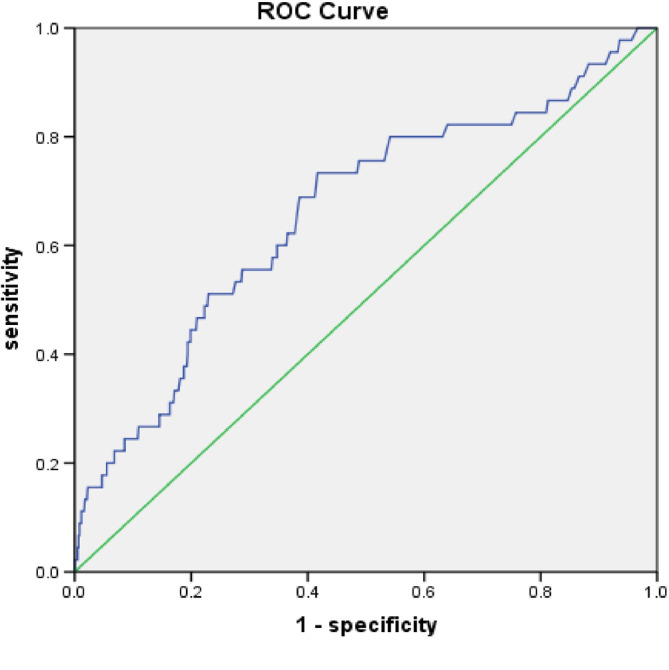
ROC curve depicting detection of new atrial fibrillation as a function of creatine kinase‐MB

To further clarify the possibility of NT‐proBNP and CK‐MB and so predict the occurrence of AF, we used ROC curves to determine a cutoff level (Figures 1 and 2). NT‐proBNP using a cutoff of 1705 ng/L resulted in a sensitivity of 58% and a specificity of 89.8%. The area under curve (AUC) for this risk score was 0.647 (CI 0.558‐0.737). CK‐MB using a cutoff of 142.5 ng/L resulted in a sensitivity of 73.3% and a specificity of 58.3%. The AUC for this risk score was 0.661 (CI 0.573‐0.749).

## 4 DISCUSSION

For patients with ACS and AF, diagnosis and treatment are difficult, especially in antithrombotic therapy, and the prognosis is very poor. The European Heart Association recommends screening for AF to reduce the incidence rate.^6^ But it is difficult to detect because most AF is asymptomatic or paroxysmal, which leads to the risk of AF being far underestimated.^7^, ^8^ Meanwhile, a considerable proportion of elderly patients with AF have not been treated.^9^

Because AF has a similar pathophysiological mechanism of occurrence and development with other cardiovascular diseases such as ischemic heart disease and heart failure, these diseases may have the same biomarkers. These biomarkers can add prognostic value for predicting the death, cardiovascular events, and heart failure of cardiovascular diseases.^10^ We incorporated all factors that might cause AF in ACS patients into the model for multivariate analysis. Only NT‐proBNP, CK‐MB, and LVEF were found significantly related with incident AF. The levels of NT‐proBNP and CK‐MB were higher and the LVEF were worse in ACS patients who subsequently developed AF. Over time, CK‐MB and NT proBNP gradually returned to normal, and LVEF was still related with incident AF. A large number of previous studies have found that NT‐proBNP or TnI are good predictors of myocardial infarction, heart failure, ischemic stroke, AF, and mortality.^11^, ^12^, ^13^ Our study did not indicate that TnI level was related to the occurrence of AF, but CK‐MB level, which is also a biomarker of myocardial necrosis, was found to predict the development of AF. It is possible that longer changes in myocardial structure resulted in more pronounced cardiac injury, and a greater chance of AF.

Using the ROC curves, the best cutoff value to predict AF occurrence at the NT‐proBNP was 1705 ng/L. The best cutoff value of CK‐MB was 142.5 ng/L. The patients with newly detected AF may have high NT‐proBNP because of the stress on the heart caused by arrhythmia. It may also be a sign of cardiac pathology, which makes the patients more prone to arrhythmia. Through the detection of NT‐proBNP and CKMB, AF can be prevented and intervention performed as early as possible to prevent the progressive deterioration of the condition. Several previous cohorts have quantified the likelihood of NT‐proBNP leading to AF,^14^, ^15^ but there have been few studies of CKMB. Our study further proves that NT‐proBNP is closely related to the development of AF and significantly improved the predictive ability, which is consistent with many previous studies.^16^ Our study also found that CK‐MB might be an important index to predict the occurrence of AF.

## 5 LIMITATIONS

Our study has some specific limitations that need to be considered. First, although all of the patients were followed up, most of the clinical data were 3 to 5 years ago, which may have some impact on the results. Second, because it is a retrospective study, we could not discern the causal relationship. We do not know whether NT‐proBNP and CK‐MB would be useful as a marker of elevated risk after AF onset or as a marker of incident AF. Finally, only ACS patients were included, and the number of patients included was small. Further large‐scale, multicenter, prospective data are needed to further confirm the research results.

## 6 CONCLUSION

For patients with ACS, NT‐proBNP, CK‐MB, and LVEF are the main prognostic indicators used to predict new onset AF. Quantitative measurement based on NT‐proBNP and CK‐MB can be used to determine the value conducive to AF screening.

## CONFLICT OF INTEREST

The authors declare no potential conflict of interest.
